# A recombinant measles virus vaccine strongly reduces SHIV viremia and virus reservoir establishment in macaques

**DOI:** 10.1038/s41541-021-00385-6

**Published:** 2021-10-22

**Authors:** Patrycja Nzounza, Grégoire Martin, Nathalie Dereuddre-Bosquet, Valérie Najburg, Leslie Gosse, Claude Ruffié, Chantal Combredet, Caroline Petitdemange, Sylvie Souquère, Géraldine Schlecht-Louf, Christiane Moog, Gérard Pierron, Roger Le Grand, Thierry Heidmann, Frédéric Tangy

**Affiliations:** 1 Molecular Physiology and Pathology of Endogenous and Infectious Retroviruses Unit, CNRS UMR 9196, Gustave Roussy, Villejuif, France; 2 UMR 9196, Université Paris-Sud, Orsay, France; 3 VIROxIS, Gustave Roussy, Villejuif, France; 4 Immunology of Viral Infections and Autoimmune Diseases, IDMIT Department, IBJF, CEA-Université Paris-Sud-INSERM U1184, Fontenay-Aux-Roses, France; 5 Viral Genomics and Vaccination Unit, Department of Virology, Institut Pasteur, CNRS UMR 3965, Paris, France; 6 UMR996-Inflammation, Chimiokines et Immunopathologie, INSERM, Faculté de médecine, Université Paris-Sud, Université Paris-Saclay, Clamart, France; 7 INSERM U1109, Fédération de Médecine Translationnelle de Strasbourg (FMTS), Université de Strasbourg, Strasbourg, France

**Keywords:** Preclinical research, Live attenuated vaccines

## Abstract

Replicative vectors derived from live-attenuated measles virus (MV) carrying additional non-measles vaccine antigens have long demonstrated safety and immunogenicity in humans despite pre-existing immunity to measles. Here, we report the vaccination of cynomolgus macaques with MV replicative vectors expressing simian-human immunodeficiency virus Gag, Env, and Nef antigens (MV-SHIV Wt) either wild type or mutated in the immunosuppressive (IS) domains of Nef and Env antigens (MV-SHIV Mt). We found that the inactivation of Nef and Env IS domains by targeted mutations led to the induction of significantly enhanced post-prime cellular immune responses. After repeated challenges with low doses of SHIV-SF162p3, vaccinees were protected against high viremia, resulting in a 2-Log reduction in peak viremia, accelerated viral clearance, and a decrease -even complete protection for nearly half of the monkeys- in reservoir cell infection. This study demonstrates the potential of a replicative viral vector derived from the safe and widely used measles vaccine in the development of a future human vaccine against HIV-1.

## Introduction

Despite the high efficacy of antiretroviral therapy (ART), AIDS still kills nearly one million people each year^1^. HIV infection remains highly active in certain populations due to limited access to treatment and lack of adherence to protective measures against sexual exposure to HIV. The development of an HIV vaccine is therefore needed more than ever. It relies primarily on the selection of the best vector/vaccine strategy. To date, a limited number of vaccine vectors have been evaluated in humans in phase 2–3 trials. Of these, canarypox used as a prime followed by a protein boost in the RV144 trial is the only one to have demonstrated some level of protection with a 31% reduction in the risk of HIV infection at 42 months^2^. However, it did not maintain immunity during the first year and had no impact on viral load or CD4 + T-cell depletion in those infected during the trial^2,3^. More importantly, its subsequent evaluation in South Africa in a phase 2b-3 trial has just been suspended due to lack of evidence for efficacy^4^.

Obviously, the development of a prophylactic HIV vaccine requires alternative vectors/vaccine strategies. Vectoring with Ad26, a non-replicating adenovirus, conferred partial protection against acquisition of infection against SHIV-SF162p3 or SIVmac251 in non-human primates (NHPs) when used in prime followed by protein boosts^5,6^. Tested in phase I/IIa trials, this vaccine candidate has been shown to be safe and immunogenic^7^. Currently, this vaccine approach is undergoing two phase 2b-3 clinical evaluations in South Africa (HVTN 705) and the United States (MOSAICO)^8^. Another live vector that persists in the host, rhesus cytomegalovirus (RhCMV), has been evaluated in non-human primates challenged with SIVmac251^9^. Although not preventing initial infection, this vectorization induced potent stimulation of SIV-specific CD8 + T cells that controlled and eliminated the virus in 50% of animals^9,10^. However, this strategy, which induces CD8 + T-cell responses limited to MHC-II or MHC-E in NHP, still needs to be adapted to humans before being tested in clinical trials^11^. A novel vaccine based on mRNA encapsulated in lipid nanoparticles evidenced by the COVID pandemic will soon be evaluated against HIV, including using antigens whose design was guided by their recognition by broadly neutralizing antibodies^12,13^.

Although used in human vaccination for decades, the measles virus (MV) has never been applied as a vaccine vector in humans against HIV. To this end, we conducted a pilot study to evaluate the protective capacity in NHPs of a prime/boost homologous immunization with MV expressing SHIV antigens. Live-attenuated measles vaccine has been safely administered to over 2 billion children during the last 40 years, affording life-long protection with an efficacy rate of 93–97% after one or two administrations^14^. We previously derived a vaccination vector from the measles vaccine and demonstrated its capacity to induce long-term protective immunity in mice and NHP against different viruses even in the presence of pre-immunity to the vector^15,16^. The strong immunity of this vector is based on MV replication, which results in the expression of antigens in vivo in antigen-presenting cells naturally targeted by the measles virus^17^. Efficient targeting of antigen-presenting cells is considerable since they are able to induce strong antibody and long-lived cellular responses that can broadly disseminate to systemic and mucosal compartments. Human proof of concept of this strategy was demonstrated for a measles-chikungunya vaccine (MV-CHIK) that we previously described^18^ and that was successfully tested in phase I and II trials^19,20^. The vaccine was well-tolerated and induced a robust and functional response in 100% of volunteers after two administrations. Importantly, pre-existing measles antibodies did not alter the immunogenicity of the heterologous antigen, confirming that pre-immunity to measles due to vaccination or infection does not restrict the use of recombinant MV for new vaccines. A MV-HIV vaccine might therefore be considered for adolescents and young adults, the main target population for HIV infection/transmission. Moreover, the MV vector can be rapidly introduced in clinical trials, and it is a cheap and easy to manufacture vaccine (price per dose in <0.3 US$, www.unicef.org).

We have previously shown that recombinant MV-HIV vaccines independently expressing HIV Env, Gag or a Gag-RT-Nef fusion protein can induce strong humoral and cell-mediated immune responses in NHP^21,22^. We had also shown that simultaneous co-expression of Gag and Env led to the formation of virus-like particles (VLPs), that proved to be very immunogenic, at least in mice^23^. Here, we therefore constructed MV-SHIV vectors to simultaneously express the SHIV Gag, Env, and Nef antigens, which we further tentatively optimized by introducing specific mutations to abolish their immunosuppressive activity and so enhance vaccine immunogenicity. Indeed, like other retroviruses^24^, HIV and SIV lentiviruses have an immunosuppressive (IS) domain in their Env protein^25^, and they also have one in the lentivirus-specific Nef protein^26^. This immunosuppressive activity can be abolished by definite point mutations that can be monitored by specific in vivo tumor rejection assays in which tumor cells expressing mutated IS domains are rapidly rejected as compared with tumor cells carrying wild-type IS domains^27^. Such mutations within the IS domain of a murine leukemia virus (the Friend-MLV) were previously shown to abolish virus resistance to the mice’s immune system^24^. Furthermore, a canarypox-vectored feline leukemia virus (FeLV) vaccine encompassing targeted mutations within the FeLV Env IS domain was demonstrated to generate increased protection against an infectious FeLV challenge in vivo and resulted in a marketed veterinary vaccine^28^.

In this study, we tested the capacity of homologous prime-boost immunization with MV-SHIV vaccine to protect *cynomolgus* macaques from a challenge with repeated intrarectal low doses of the difficult to neutralize SHIV-SF162p3. We show that MV-SHIV vaccination reduced challenge virus infection by a hundred-fold, rapidly controlled its propagation, and limited cell-reservoir establishment resulting in 50% of animals with undetectable viral and proviral load. We also show that the targeted mutations in the Nef and Env IS domains in the MV-SHIV vaccine significantly increased cellular immune responses. Altogether these results demonstrate the value of measles vector-based vaccine strategies, and provide promising issues for the control of HIV infection in humans.

## Results

### Vaccine vectors and NHP study design

**Fig. 1:**
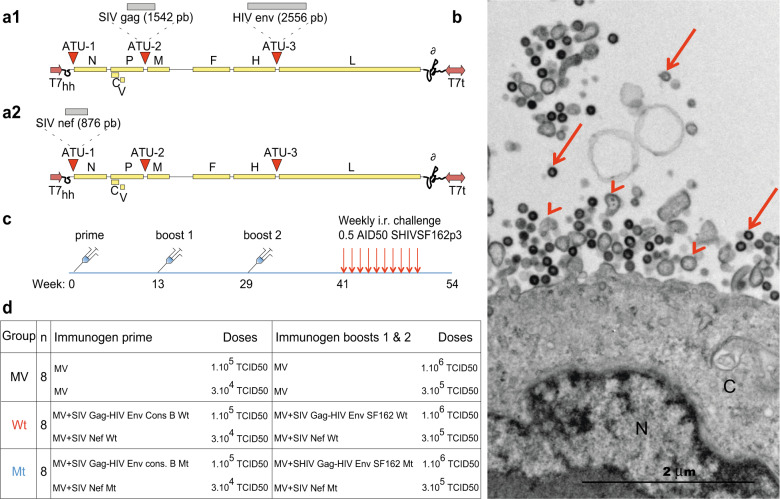
Vectors, VLP electron microscopy, and vaccine regimen. **a1** pMVSchw vector with ATU2 containing SIV gag gene and ATU3 containing HIV env gene WT or mutated. HIV env genes are HIV env cons B subtype for the prime vector and SF162 subtype for boosts 1 and 2. **a2** pMVSchw vector with ATU1 containing SIV nef gene WT or mutated. HIV env and SIV nef genes were mutated at their immunosuppressive domains. The MV genes are indicated as follows: N (nucleoprotein), P (phosphoprotein), V and C proteins, M (matrix), F (fusion), H (hemagglutinin), L (polymerase), T7 (T7-RNA polymerase promoter), T7t (T7-RNA polymerase terminator), δ (hepatitis delta virus ribozyme). **b** Electron microscopy image of Vero cells infected by recombinant MV-SHIV Wt virus (MOI of 0.01, MV-SIV Gag-HIV Env). N nucleus, C cytosol. Arrowheads indicate MV viral particles and arrows gag-forming VLPs. **c**, **d** Summary of vaccine: immunization schedule, and repeated low dose of intrarectal SHIVSF162P3 challenges. Prime and boost 1 immunizations were subcutaneous and boost 2 was both subcutaneous and intranasal. Subcutaneous inoculations were performed at two distant sites in the back of animals.

To evaluate the capacity of MV vaccine vectorization combined with IS domain mutations of the antigens, we generated MV-SHIV vectors simultaneously expressing Gag Env or Nef. The sequences corresponding to SIVmac239 gag and HIV-1 env genes were inserted into two distinct additional transcription units (ATU) of MV vector (consensus B Env for prime and SF162 Env for boosts) (Fig. 1a1). Another MV vector was generated expressing SIVmac239 Nef as a secreted protein (Fig. 1a2). HIV Env or SIV-Nef IS domain mutants were defined based on the ability of cells transduced with these mutated antigens to be rejected in mice compared to cells transduced with the wild-type forms, according to previously described in vivo assays (Supplementary Fig. 1A, B)^25–27^. We had also previously shown that the co-expression of Gag and Env proteins formed very immunogenic virus-like particles (VLPs)^23^. Electron micrographs of Vero cells infected with the present MV-SHIV vaccine virus evidenced the production of both MV particles and Gag-assembled VLPs (Fig. 1b). Furthermore, the Gag, Env, and Nef antigens carried by measles viruses were properly expressed, as verified by western blot analysis (Supplementary Fig. 2).

We immunized 24 *cynomolgus* macaques subcutaneously with three injections (at weeks 0, 13, and 29) of MV vectors expressing SHIV antigens in their wild type or IS domain mutant forms or no antigen (control, MV) (Fig. 1c, d). The subcutaneous route was preferred to the intramuscular route for consistency with our previous studies using measles virus as a vaccine vector^29,30^. MHC haplotypes of animals were distributed in the different groups, and three animals that carried the H6 haplotype known to be associated with increased control of HIV/SIV^31^ were equally distributed in the three groups. To evaluate the protective efficacy of the different vaccine regimens, 3 months after the last immunization (week 41) all animals were challenged by repeated intrarectal administrations once a week with 0.5 AID50 of tier-2 SHIV-SF162p3 (a dose estimated to infect 25% of animals at each challenge, Fig. 1c). Plasma viral load was assessed every week by qRT-PCR and challenges were stopped after two subsequent virus detections in plasma.

### Vaccination with MV-SHIV protects from high viremia upon repeated SHIV-SF162p3 challenges

**Fig. 2:**
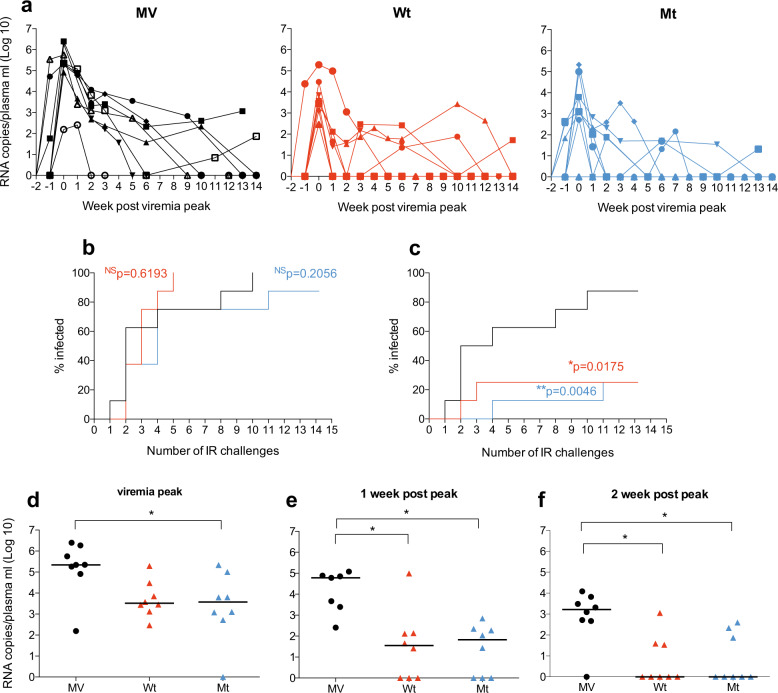
Control of SHIV-SF162p3 replication in immunized animals. MV: control, Wt: MV-SHIV Wt, Mt: MV-SHIV Mt. **a** Longitudinal study of plasma viral load in control (MV) and vaccinated (Wt and Mt) animals starting from the week “viremic peak -2”. **b** Percentage of infected animals after each challenge (no significant difference between the three groups). **c** Percentage of infected animals after each challenge with plasma viral load above 10^4^ virus copies/mL (**b** and **c** Kaplan–Meyer plots; *P* values are calculated by the log-rank Mantel–cox test). **d** Peak plasma viral load. **e** Plasma viral load at 1 and (**f**) 2 weeks post peak (*P* values are calculated by Kruskal–Wallis and Dunn’s multiple comparisons tests). Group medians are plotted as a horizontal line. **b**–**f** *P* values: NS > 0.05, *< 0.05, **< 0.01.

Although all animals (except one in the mutant group) were finally infected after 10 weeks of SHIV-SF162p3 repeated challenge with no significant deviation from control for the onset of infection (Fig. 2a, b), vaccination with MV-SHIV protected from high viremia. Indeed, 75% of vaccinees exhibited low virus levels below 10^4^ virus copies/mL at peak of infection (*P* = 0.0175 for MV-SHIV Wt and *P* = 0.0046 for MV-SHIV Mt, log-rank Mantel–Cox test, Fig. 2c). SHIV-SF162p3 plasma viremia was reduced by 2-log at peak in vaccinated animals (*P* < 0.05, MV-SHIV Mt compared to MV controls, Kruskal–Wallis and Dunn’s multiple comparisons tests, Fig. 2d), and 3-log at 1 week after the peak (*P* < 0.05, MV-SHIV Wt and Mt compared to MV, Fig. 2e). At 2 weeks after peak, 10 out of 16 vaccinated animals had completely controlled the challenge virus and were negative for plasma viral load, indicating that MV-SHIV vaccination had a strong impact on the kinetics of plasma viremia clearance (*P* < 0.05, MV-SHIV Wt and Mt compared to MV, Fig. 2f). Of note, the four animals from the vaccine groups and the one from the control group that showed the lowest peak viremia (<10^3^ copies/ml) presented all a different MHC haplotype and not the H6 one previously described to be associated with a potential protection^31^.

**Fig. 3:**
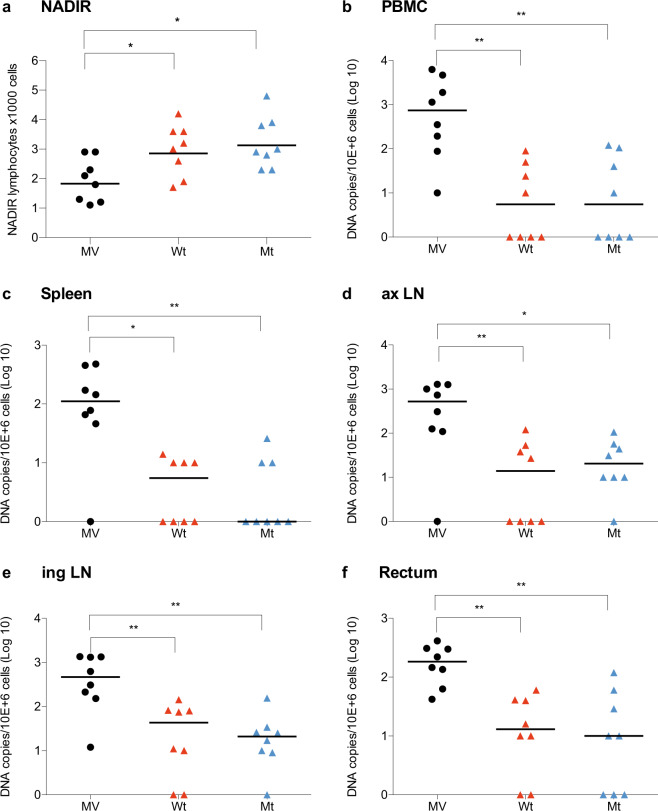
MV-SHIV vaccine protects from lymphocyte depletion and limits DNA proviral reservoir seeding. **a** Blood lymphocyte NADIR after challenge (group Geomeans are plotted as a horizontal line; *P* values are calculated by Kruskal–Wallis and Dunn’s multiple comparisons tests). **b**–**f** At necropsy (week 13 or > ) SHIV DNA copies per 10^6^ cells in PBMC (**b**), spleen (**c**), axillary (**d**), and inguinal (**e**) lymph nodes, and rectum (**f**). MV: unmodified MV vector, Wt: MV-SHIV Wt, Mt: MV-SHIV IS domain mutants. Group medians are plotted as a horizontal line; *P* values are calculated by Kruskal–Wallis and Dunn’s multiple comparisons tests. *P* values:  NS > 0.05, *< 0.05, **< 0.01.

Vaccination with MV-SHIV also protected the animals from the lymphocyte depletion that is observed in control monkeys in the period following the viral peak (lymphocyte nadir, *P* < 0.05, Kruskal–Wallis and Dunn’s multiple comparisons tests, Fig. 3a). In addition, the early control of plasma viremia in vaccinated animals resulted in a significant reduction of SHIV-SF162p3 proviral DNA in reservoirs as determined in organs and blood collected at 13 weeks after virus peak. While proviral DNA was detected in most organs of all control animals, 50% of vaccinated animals were negative in PBMCs (i.e., below the limit of detection of ten DNA copies per million cells, *P* < 0.01 Kruskal–Wallis and Dunn’s multiple comparisons tests, Fig. 3b) and 56% negative in the spleen (*P* < 0.05 for MV-SHIV Wt compared to MV and *P* < 0.01 for MV-SHIV Mt compared to MV, Fig. 3c). Virus control was less efficient in axillary lymph nodes with only 31% negative animals (*P* < 0.05 and *P* < 0.01, Fig. 3d), in inguinal lymph nodes with 25% negative animals (*P* < 0.01, Fig. 3e), and in rectum with 25% animals negative (*P* < 0.01, Fig. 3f). Furthermore, vaccinated animals that remained positive in reservoir cells had, on average, a 10–100-fold lower proviral DNA than controls.

### Breadth of MV-SHIV immune responses

**Fig. 4:**
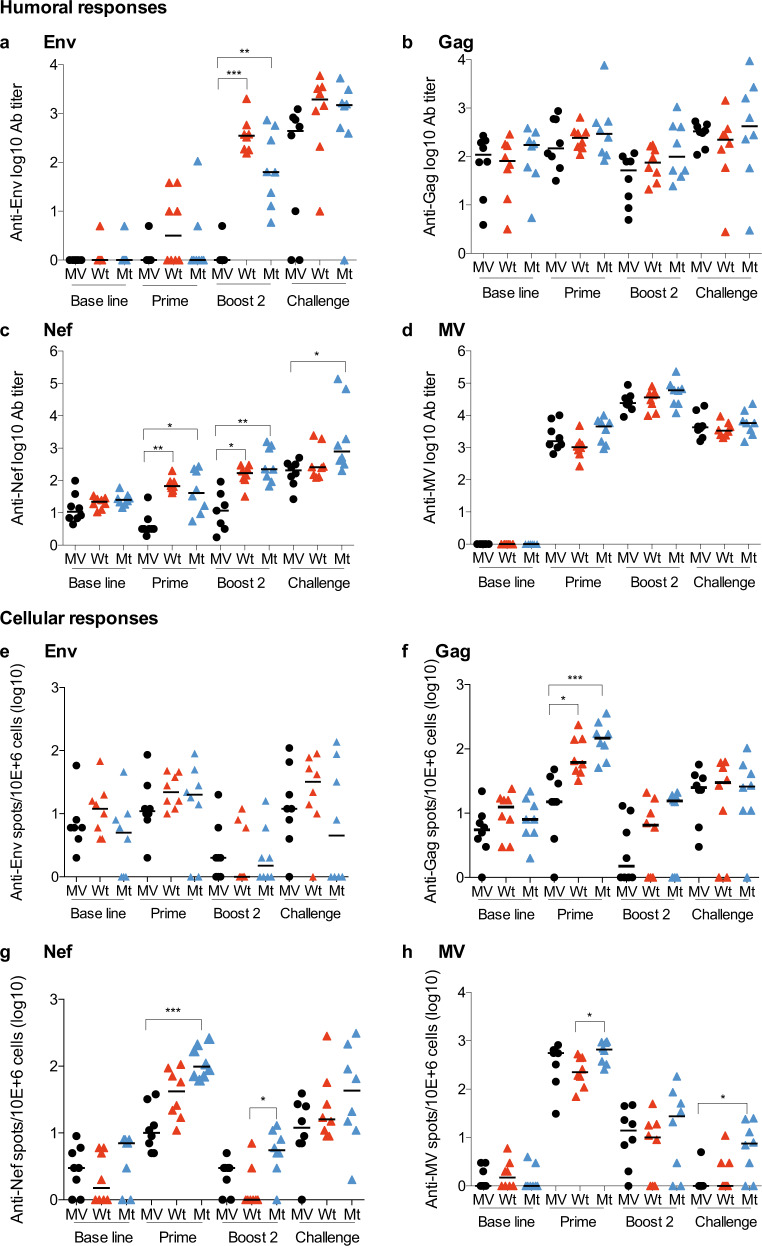
Vaccine-elicited humoral and cellular immune responses. **a**–**d** IgG antibody titers (log-endpoint ELISA titration) against (**a**) Env (gp120), **b** Gag, **c** Nef, **d** MV proteins. **e**–**h** FluoroSpot assays, IFN-γ-producing cells specific to (**e**) Env, **f** Gag, **g** Nef, and (**h**) MV proteins. *P* values are calculated by the Kruskal–Wallis and Dunn’s multiple comparisons tests. *P* values: NS  > 0.05, *< 0.05, **< 0.01, ***< 0.001. Group medians are plotted as a horizontal line. Serum or PBMC was collected at the baseline (week −2), prime (week + 2), boost 2 (2 weeks post boost 2: week +31), and post challenge (2 weeks post first positive qRT-PCR for SHIV162p3 RNA in plasma).

We next analyzed the humoral responses elicited by MV-SHIV immunizations. Antibody levels increase after each immunization (prime, boost 1 and 2; Fig. 4a, boost 1 not shown). We detected high levels of antibodies to MV, moderate to HIV Env, and low or even undetectable -close to baseline- to SIV Nef and Gag (Fig. 4a–d). Anti-Env antibody responses increased after prime and each boost to reach titers ranging from 10^2^−10^3^ after boost 2 (Fig. 4a). Anamnestic antibody responses to HIV Env were observed after challenge in animals immunized with wild-type and mutant MV-SHIV vaccines, as demonstrated by a five times increase (Fig. 4a). It is of note that post-boost levels of anti-Env antibodies in vaccinated monkeys are not higher than levels induced in controls by the challenge, which could explain the insufficient protection to virus acquisition in vaccinated animals. Borderline levels of antibodies to SIV Gag were elicited by immunization and did not increase after the challenge (Fig. 4b). Antibodies to SIV Nef were induced in all vaccinated animals after boosting and were slightly increased by the challenge (Fig. 4c). All animals had comparable levels of antibody to measles virus vector (Fig. 4d), which were increased ten times by boosting, indicating that all animals received an equal amount of vaccine vector that replicated identically. A few animals displayed low antibody-neutralizing activity after the second boost against the tier-1 HIV-1-SF162 strain, but no neutralizing activity was found against the tier-2 SHIV-SF162p3 strain (Supplementary Table 1).

We evaluated cell-mediated immune responses by IFN-γ FluoroSpot assay in response to HIV Env, SIV Gag, SIV Nef, and MV vector antigens (Fig. 4e–h and Supplementary Fig. 3A–D). HIV-Env cellular responses were low and only observed after prime with no increase after challenge (Fig. 4e and Supplementary Fig. 3A). In contrast, Gag and Nef cellular immune responses were significantly induced by MV-SHIV prime (Fig. 4f, g). Boosting did not improve these responses similarly to MV-specific cellular responses (Fig. 4f–h and Supplementary Fig. 3B–D with additional time points). Post-prime cellular responses elicited to SHIV and MV antigens waned over time despite two booster immunizations and challenge (pre-challenge cellular responses were undetectable, and Supplementary Fig. 3A–D). We previously made the same observation in macaques and demonstrated that although memory T-cell responses to MV vector are hardly detectable in circulating PBMCs and necessitate long in vitro proliferation to be detected, they persist in the secondary lymphoid organs such as lymph nodes and spleen^21,22^. The absence of boosting of T-cell responses characterizes measles virus, as shown by the same absence of boost observed for measles cellular responses after boost 2 compared with the prime (Fig. 4h and Supplementary Fig. 3D). However, this does not affect the efficacy of the measles vaccine, and it is likely that it should not affect the efficacy for any antigen carried by the measles vector, as we previously demonstrated with a measles virus-based Lassa vaccine that confers complete protection of NHP after a single shot^29,30^.

### Mutations of the IS domains increased the specific cellular responses

When we compared the cellular responses induced by MV-SHIV vaccines expressing wild-type (Wt) or mutated Env and Nef IS domains (Mt), we observed significantly higher IFN-γ responses against Gag and Nef for Mt as compared to controls (Fig. 4f, g), and against MV for Mt as compared to Wt (Fig. 4h). Post-prime IFN-γ responses to Gag and Nef in Fig. 4f and g showed that IS domain mutant antigens elicited increased responses relatively to MV controls (*P* < 0.001, Kruskal–Wallis and Dunn’s multiple comparisons tests), and as compared to wild-type antigens (*P* < 0.05 for Gag and not significant for Nef). Similarly, cellular response analysis by intracellular staining (ICS) indicated that post-boost responses for IS domain mutant antigens were significantly increased as measured by CD4 + T-cell IL-2 levels in response to Gag or CD8 + T-cell IL-2 in response to Nef (*P* < 0.01 for Gag and *P* < 0.05 for Nef, Supplementary Fig. 4A, B). Noteworthy, no significant difference was found between antibody levels elicited by IS domain mutant vaccine as compared to wild-type vaccine (Fig. 4a–d), suggesting a specific modulation of the CD4 and CD8 T-cell responses by IS domain-mutated Env and Nef antigens, as previously demonstrated for the FeLV IS domain-mutated vaccine^28^.

### Immune correlates of challenge virus control and eosinophilic status of animals

**Fig. 5:**
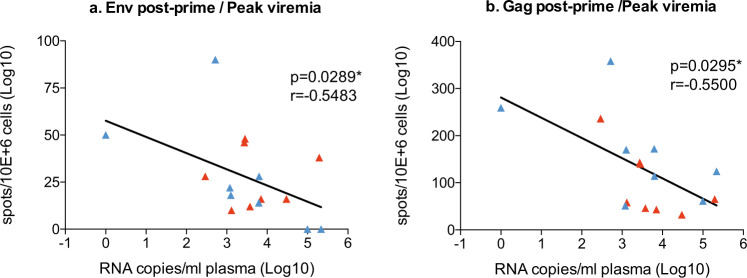
Correlation between SHIV viremia control and cellular immune responses. **a**, **b** Correlation measurements: plotted data of log SHIV RNA copies/mL at viremic peak with post-prime IFN-γ-producing cells specific to Env (**a**), Gag (**b**). FluoroSpot assays in vaccinated animals (MV-SHIV Wt and Mt). Statistical analyses are performed with the nonparametric Spearman correlation, two-tailed p-value. NS *P* > 0.05, *P < 0.05.

When comparing the magnitude of the cellular immune responses induced by MV-SHIV immunizations and the challenge virus levels measured at peak or in the following weeks, we identified some correlates of vaccine efficacy. We found that the number of post-prime anti-Env and Gag SHIV-specific IFN-γ-producing circulating T cells was inversely correlated with the peak plasma viremia (anti-Env, *P* = 0.0289, *r* = −0.5483 and anti-Gag, *P* = 0.0295, *r* = −0.5500, nonparametric correlation of Spearman, two-tailed *P* value, Fig. 5a, b). No significant correlation was found with post-prime anti-Nef and MV-specific IFN-γ-producing T cells nor with SHIV antibody titers (not shown). Of note, we found a correlation between the number of eosinophilic blood cells and the level of viremia at peak (*P* = 0.0062, *r* = 0.6647, Spearman test) (Supplementary Fig. 5). However, even the higher levels of viremia observed at peak in animals with high eosinophilia dropped dramatically in the following week in vaccinated animals, suggesting that high levels of eosinophil may impact SHIV infection outcome but not vaccine control in the first week of infection. Indeed, although mechanisms are not yet understood, a prevalence rate of 70% of eosinophilia in SIV-infected macaques compared to 10% in naive monkeys was previously reported^32^.

## Discussion

HIV vaccine vectors have been extensively evaluated in numerous animal experiments and clinical trials^4,7,33,34^. Because of their replicative nature, live vaccine vectors produce antigens over a longer period of time, resulting in strong innate and adaptive immune responses that allow early control of HIV viremia and prevent high levels of replication and immune escape^35,36^. In this study, NHP were vaccinated with MV vectors expressing SHIV Gag Env and Nef vaccine antigens and then challenged rectally by tier-2 SHIV-SF162p3. In previous work, we had shown that the immunity induced by MV vaccine vector depends on its replication^37^. In addition, MV infection experiments in macaques reported that macrophages and dendritic cells are the main targets of the virus during the first week of infection, which would contribute to high viral antigen presentation and the induction of multi-epitopic responses^17^. Vaccination studies in *rhesus* macaques with the live cytomegalovirus vector expressing SIV antigens (CMV-SIV) have reported the maintenance of specific cellular immune responses over a long period of time and the induction of differentiated effector cells at early SIVmac239 replication sites^9–11^.

In this pilot study, we show that vaccination with live-attenuated MV expressing SHIV antigens did not significantly affect the number of SHIV-SF162p3 challenges required to achieve infection relative to control-vaccinated animals, but very strongly reduced the peak challenge viremia and accelerated plasma viral clearance. This early control of SHIV-SF162p3 infection resulted in a significant reduction of the size of proviral reservoirs in secondary lymphoid organs and rectal mucosa. Unlike control-vaccinated animals, at least 50% of MV-SHIV-vaccinated animals showed an absence of integrated virus or less than 10 proviral copies per million cells. Moreover, vaccinated animals that remained positive in reservoir cells had-on average-a viral load 10–100 times lower than control vaccines.

SHIV-SF162p3 infection is naturally controlled 13 weeks post challenge in the majority of *cynomolgus* macaques. Vaccination with MV-SHIV accelerated this process by reducing to less than to 2 weeks the time to control, which is consistent with immunological control before the amplification and systemic spread of the virus. The immune responses elicited to MV-SHIV vaccination thus afforded a significant control of viremia, which is of particular importance in the perspective of a vaccine applicable to humans and in view of the viral control observed in certain patients, which preserve them from AIDS^38,39^.

In the SIV infection model (i.e., “uncontrolled infection”), vaccination with CMV-SIV induces immunological clearance of reservoir cells in 50% of monkeys after demonstrated systemic viral dissemination^9,10^. The importance of controlling and reducing viral integration into the lymph nodes has been highlighted in a recent study comparing non-pathogenic and pathogenic SIV infections in primates^40^. Control of infection in rectal mucosal cells and lymph nodes induced by MV-SHIV vaccination suggests the induction of specific immune responses at the first sites of virus replication. This could be explained by the induction of the strong anti-Gag-specific cellular responses we obtained, which have previously been shown to control viral infection in non-human primates^41^. Several studies have reported protection from SHIV-SF162p3 following mucosal vaccination of NHP^42,43^. It would be desirable to continue the evaluation of the MV-SHIV in a vaccination protocol aimed at inducing strong mucosal immunity through the administration of a prime followed by a series of mucosal boosts.

In addition, MV-SHIV vaccine protected against lymphocyte depletion. This protection can be related to the correlation that we observed between T-cell-specific responses and viremia control. The protective role of cellular immune responses has already been described in primates exposed to SHIV challenges^44,45^ or in humans infected with HIV-1^46^. Although we did not find a correlation between vaccine antibody titers and viral load levels, we cannot exclude that they contributed to the control of viremia, as previously shown in NHP-infection model through viral entry neutralization or antibody-dependent cytotoxicity/phagocytosis mechanisms^47,48^. We were unable to detect IgA or IgG mucosal antibodies in rectal secretions and our results indicate the absence of neutralizing antibodies against SHIV-SF162p3. Although we did not perform an analysis of innate immunity in this pilot experiment, we know from a previous study performed with a MV-LASV vaccine^29^ that the early changes induced in immunity pathways as early as two days after MV vaccination, such as type I/II IFN responses, inflammatory responses, antigen presentation, and T-cell responses, also correlate with protection from challenge.

Our study also aimed to evaluate the effects of mutations in the IS domains of the Env and Nef antigens. Interestingly, these mutations induced higher cellular immune responses in the vaccinees, as similarly observed-although to a larger extent-for the FeLV vaccine in which we had previously inactivated the Env IS domain by point mutation^28^. The mutated vaccine antigens also resulted in slightly reduced proviral loads after the challenge of the vaccinees with SHIV-SF162p3, but the differences in protection remained limited, with the exception of one in eight animals in the mutant regimen that remained virus-free after 13 subsequent rectal viral challenges. Of note, the enhancing effect of the IS mutations appears to be most visible on the immune response after prime immunization, and the mutated antigens might therefore have a clearer benefit for protection in a single dose and/or alternative vaccination regimen.

Vaccine protection against SHIV-SF162p3 resistance to neutralization has been achieved only in a limited number of NHP trials^6,7,42,43^. CCR5-tropic SHIV-SF162p3 is a stringent NHP challenge model that induces a very low level of neutralizing antibody in long-term-infected NHP^49^. In addition, despite their homology, vaccination with Env SF162 does not induce protective immunity against SHIV-SF162p3 or viremia control^50^. This suggests that the control of viremia induced by the present MV-SHIV vaccine is likely not attributable to the sole utilization of Env SF162 in the two boosts.

Even if non-fully protective, MV-HIV vaccination in humans could delay or even prevent the subsequent onset of AIDS. Such a vaccination strategy could be compared to active antiviral treatments (ART) administered a few hours or days after infection that have proven their therapeutic effectiveness^51,52^. Of particular interest, broadly neutralizing antibodies treatments of NHP infected by the SHIV AD8-EO elicited strong cellular responses that correlated with virus control, leading to undetectable plasma viremia in half of the monkeys, while under the same conditions ART early administration did not prevent sustained viral rebounds following treatment interruption^53^. In addition, vaccination that would allow patients to move from progressor to controller status would have an important impact on secondary transmission, since 50% of human infections occur in donors who are in an acute or early stage of infection^54,55^. Finally, human MV-HIV vaccination due to its long-term antiviral immunity may limit the establishment of viral reservoirs against which there is no effective treatment to date.

This study is the first demonstration that a measles-derived replicating vaccine vector is able to provide some protection from high viremia and reservoir establishment in NHP. Virus control was achieved with no need for heterologous boosting or complex vaccine composition or combinations, and correlated with levels of cellular immune responses. Mutations of IS domains clearly increased vaccine immunogenicity, indicating that they should be included in other HIV vaccine strategies. The measles vaccine platform has already demonstrated clinical feasibility. It is a cheap live vaccine easy to manufacture and to distribute. Preventing AIDS with a pediatric vaccine would be an ideal goal, and clinical studies are now required to determine the potential of such MV-HIV-1 vaccine candidate in humans either alone or combined with an adjuvanted protein boost -including mucosal vaccination- with the aim of inducing enhanced neutralizing antibody responses.

## Methods

### Ethics statement

Macaques (*Macaca fascicularis*) were housed in the facilities of the Infectious Disease Models and Innovative Therapies (IDMIT) centre, part of the “Commissariat à l’Energie Atomique et aux Energies Alternatives” (CEA, Fontenay-aux-Roses, France). Non-human primates (NHP) were used at the CEA in accordance with French national regulations and under the supervision of national veterinary inspectors (CEA Permit Number A 92-032-02). The CEA complies with the Standards for Human Care and Use of Laboratory Animals, of the Office for Laboratory Animal Welfare (OLAW, USA) under OLAW Assurance number #A5826-01. The use of NHP at the CEA is in conformity with the recommendations of the European Directive (2010/63, recommendation N°9). The animals were used under the supervision of the veterinarians in charge of the animal facility. This study was approved and accredited under statement number A14-042 by the ethics committee “Comité d’Ethique en Expérimentation Animale du CEA” registered under number 44 by the French Ministry of Research. Animals were housed in adjoining cages allowing social interactions, under controlled conditions of humidity, temperature, and light (12-h light/12-h dark cycles). Water was available ad libitum. Animals were monitored and fed 1–2 times daily with commercial monkey chow and fruits by trained personnel. Macaques were provided with environmental enrichment including toys, novel foodstuffs, and music under the supervision of the CEA Animal Welfare Body. Experimental procedures (animal handling, immunizations, intrarectal inoculations, blood, and lymph node samplings) were conducted after animal sedation with ketamine chlorydrate (Rhône-Mérieux, Lyon, France, 10 mg/kg). Tissues were collected at necropsy: animals were sedated with ketamine chlorhydrate 10 mg/kg) then euthanized by intravenous injection of 180 mg/kg sodium pentobarbital.

### Plasmids constructions and vectors production

The plasmid pTM-MVSchw carries an infectious cDNA corresponding to the anti-genome of the Schwarz MV vaccine strain^23^. Additional transcription units (ATU1-3) have been inserted into the plasmid backbone by site-directed mutagenesis between definite MV genes. Each MV open-reading frame (ORF) expression is controlled by its own cis-acting element. The expression of additional ORFs inserted in an ATU is controlled by *cis*-acting elements modeled after those present in the N/P boundary region (allowing for the necessary transient transcription stop upstream of the transgene, autonomous transcription, capping, and polyadenylation of the transgene). Into a single pTM-MVSchw plasmid: SIVmac239 Gag and HIV-1 Env (Consensus B Env for the prime and SF162 Env for the boosts^23^) genes have been sub-cloned in the ATU2 and ATU3 respectively (Fig. 1a1). Into another pTM-MVSchw plasmid: SIVmac239 Nef gene has been sub-cloned into ATU1 (Fig. 1a2). SIVmac239 Nef gene encodes for a secreted and non-myristoylated form. The corresponding viruses were rescued from the pTM-MVSchw-SHIV plasmids using a helper cell-based system. Briefly, helper HEK293 cells expressing both the T7-RNA polymerase and the Schwarz MV N and P proteins (HEK293-T7-MV) were co-transfected with the pTM-MVSchw-SHIV (either encoding for Gag Env or Nef antigens) and a plasmid expressing the Schwarz MV polymerase L. Subsequently, transfected HEK293-T7-MV helper cells were gently harvested and co-cultured with Vero cells for the amplification of the MVSchw-SHIV viruses. Virus titers were determined by endpoint titration on Vero cells and expressed as TCID_50_/ml. Briefly, Vero cells were seeded into 96-well plates (7500 cells/well) and infected by serial 1:10 dilutions of virus sample in DMEM–5% FCS. After incubation for 7 days, cells were stained with crystal violet and the TCID50 values were calculated by use of the Kärber method (1931). Replication of each MV-HIV recombinant vector on Vero cells was analyzed by single-step growth curves using an MOI of 0.01. The growth rate of the recombinant MV carrying the SHIV Gag and Env antigens was lower than those with Nef, possibly associated with the reduced length of the later antigen, so a threefold lower dose of MV-Nef viruses was co-administered for each immunization.

### Western blotting

Vero cells were infected with the MV-SHIV virus at an MOI of 0.1. Infected cells were collected 48 h later and lysed with complete EDTA-free protease inhibitor (Roche) in 120 mM NaCl solution. Protein lysates were separated (50 ng per well) by SDS-PAGE electrophoresis on 4–12% NUPAGE^®^ Bis-Tris gels with MES running buffer (Invitrogen). To detect the SHIV antigens (all provided by the USA NIH HIV reagent program, unless otherwise specified) the membranes were blotted with MA1-71522 for SIV Nef (Thermofisher Scientific); 2F12 for SIV Gag, F240 for HIV gp41. A sheep anti-mouse IgG-horseradish peroxidase (HRP) conjugate (NA931V; GE Healthcare) or anti-human IgG-HRP conjugate (NA933V, GE Healthcare) was used as the secondary antibody. Peroxidase activity was visualized with an enhanced chemiluminescence detection kit (Pierce).

### Identification of HIV Env and SIV nef immunosuppressive (IS) domain mutations by tumor rejection assays

293T cells (7.5 × 10^5^) were co-transfected with HIV *env or nef* gene fragments point-mutated or not at the IS domain inserted into pDFG retroviral vectors (1.75 μg) and expression vectors for the MLV proteins (0.55 μg for the amphotropic MLV *env* vector and 1.75 μg for the MLV *gag* and *pol* vector)^27^. Thirty-six hours after transfection, supernatants were harvested for infection of MCA205 cells (2.5 ml per 5 × 10^5^ cells with 8 mg/mL polybrene). Cells were maintained in selective medium (400 units/ml hygromycin) for 3 weeks and then washed with PBS, scraped without trypsinization, and inoculated s.c. in mice flanks. Tumor area (mm^2^) was determined by measuring perpendicular tumor diameters, and extent of immunosuppression was quantified by an index based on tumor size (A_IS domain_ − A_none_)/A_none_, where A_IS domain_ and A_none_ are the mean areas at the peak of growth of tumors from mice injected with Env or Nef IS domain-expressing or control cells, respectively. Mice were maintained in the animal facility of Gustave Roussy Institute in accordance with institutional regulations.

### Transmission electron microscopy

MV-SHIV infected cells fixed in 1.6% glutaraldehyde in 0.1 M phosphate buffer were collected by scraping and centrifuged. Cell pellets postfixed with 2% osmium tetroxide were dehydrated in ethanol and embedded in Epon™ 812. Ultrathin sections stained with standard uranyl acetate and lead citrate solutions were observed under a FEI Tecnai 12 electron microscope. Digital images were taken with a SIS MegaviewIII CCD camera.

### Animals, immunizations, challenge

In total, 24 naive male *cynomolgus* macaques (CM) *(Macaca fascicularis*), each weighing 4–5 kg, imported from Mauritius were assigned in the study. Animals were confirmed negative for SIV, STLV (simian T-lymphotropic virus), herpes B virus, filovirus, SRV-1, SRV-2a (Simian retrovirus 1 and 2a), and MV. Eight animals were assigned per group of immunization (Fig. 1d). The three animals carrying the H6 MHC class I haplotype were equally distributed among the three experimental groups (one macaque per group)^31^. (i) Group “MV”: animals were immunized with MV empty vector as a control, (ii) group “Wt”: animals were immunized with MV vectors encoding wild-type SIV Gag and HIV Env and wild-type Nef, and (iii) group “Mt”: animals were immunized MV vectors encoding wild-type SIV Gag, IS domain-mutated HIV Env and SIV Nef. Prime was performed with wild type or IS domain mutant consensus B Env, and boost 1 and boost 2 with Wt or Mt full-length SF162 Env.

Vaccine vectors were injected subcutaneously at weeks 0, 13, and 29. MV, MV-SHIV Wt or Mt encoding for Gag and Env proteins were injected at 1.10^5^ 50% Tissue Culture Infective Dose (TCID50), and MV-SIV-Nef Wt or Mt at 3.10^4^ TCID50. Boosts 1 and 2 were performed with a tenfold increased dose regarding the prime (1.10^6^ TCID50 MV-SHIV Gag Env Wt/Mt and 3.10^5^ TCID50 MV SIV-Nef Wt/Mt). Boost 2 was administered both subcutaneously and intra-nasally: each animal received 1 × 10^6^ MV-SHIV Gag Env Wt/Mt and 3 × 10^5^ MV-SIV-Nef Wt/Mt TCID50. For the challenges, macaques were repeatedly injected once weekly by the intrarectal route with 0.5 Animal Infectious Dose 50% (AID50) of SHIV-SF162p3. The SHIV-SF162p3 stock was provided by the NIH AIDS Research and Reference Reagent Program (SHIV-SF162p3 Virus, P070 derived, Harvest 2 1.26.09) and used as supplied. Prior to the study, this virus stock was assessed in vivo in cynomolgus macaques exposed by the intrarectal route. The titer of the stock for intrarectal exposure was 30 AID50/mL. Plasma viral loads were measured weekly and challenges were pursued until two consecutive qRT-PCR virus detections, with a maximum of 13 inoculations.

### Plasma virus and provirus quantification

Plasma SHIV RNA was quantified as previously described^56,57^. The lower limit of quantification (LOQ) and the lower limit of detection (LOD) were 37 and 12.3 copies of vRNA/mL, respectively. SHIV DNA copy numbers were measured in PBMC and organs by quantitative real-time PCR. PCR was performed in duplicate on 500 ng of DNA using SIV *gag* primers and probe as described above and on 50 ng of DNA for *GAPDH* gene using primers F 5’-ATGACCCCTTCATTGGCCTC-3’, R 5’-TCCACGACATACTCAGTGCC-3’, probe FAM-5’-CGAGCTTCCCGTTCTCAGCC-3’-BHQ1. The *GAPDH* gene was used to normalize results per million cells using a standard curve of DNA considering that 1 µg of DNA corresponds to 131,300 cells. SIV *gag* standard curve was generated by dilution of pCR4-TOPO-SIVmac251 *gag* cDNA in DNA of lymph nodes of SHIV-negative macaques. SHIV DNA copy numbers were calculated by interpolating CT of samples in *gag* standard curve and were normalized using *GAPDH* gene data to be expressed per million cells. The limit of detection is ten copies per million cells. Measurements were performed at week +13 post first SHIV detection in plasma.

### FluoroSpot IFN-γ and IL-2 assays

IFN-γ and IL-2 responses were analyzed in PBMC by using FluoroSpot assay (FS-2122-10 Monkey IFN-γ/IL-2 FluoroSpot kit from Mabtech, Nacka, Sweden) according to the manufacturer’s instructions. The following peptide pools were used for ex vivo stimulation (2 µg/mL): Gag-SIVp15-p27 (15 mers, provided by Proteogenix) in 1 pool of 85 peptides; Nef-SIV (15 mers, provided by Proteogenix) in 1 pool of 63 peptides; HIV-1 Consensus B Env peptides Complete Set (15 mers, provided by NIH, cat. #9480), divided into 3 pools of 70 peptides and MV Schwarz virus (1 pfu/cell). PMA/ionomycine were used as a positive control. Plates were incubated for 44 h at +37 °C in an atmosphere containing 5% CO_2_. Spots were counted with an automated FluoroSpot Reader ELRIFL04 (Autoimmun Diagnostika GmbH, Strassberg, Germany). Finally, the spot counts obtained for each peptide pool incubation were subtracted from those for the DMSO solvent condition.

### Intracellular cytokine assay (ICS)

In total, 2 × 10^6^ PBMCs were incubated in 200 μL of complete media (RPMI 1640 with l-glutamine containing 10% fetal calf serum FBS) with anti-CD28 (1 μg/mL) and anti-CD49d (1 μg/mL) (BD Biosciences, San Diego, CA, USA). Brefeldin A (Sigma-Aldrich, Saint Louis, MO) were added to each well at a final concentration of 10 μg/mL, and plates were incubated at 37 °C, 5% CO_2_ overnight and different conditions for stimulation were applied: (i) DMSO solvent as control, (ii) HIV Env peptide pool (2 μg/mL), (iii) SIV-Nef peptide pool (2 μg/mL), (iv) SIV Gag peptide pool (2 μg/mL), (v) MV Schwarz virus (1 pfu/cell), (vi) SEB as positive control (4 µg/mL). After washing in staining buffer, cells were stained with a viability dye (violet fluorescent reactive dye, Invitrogen), and then fixed and permeabilized with the BD Cytofix/Cytoperm reagent. Permeabilized cell samples were stored at −80 °C before the staining procedure with the following antibodies: CD3, CD4, and CD8 (used as lineage markers), and IFN-γ, TNF-α, IL-2, and CD154. After incubation, cells were washed in BD Perm/Wash buffer before being resuspended in 200 μL of wash buffer and acquired with the BD Canto II Flow Cytometer (BD Biosciences). Flow Cytometry data were analyzed using Flowjo software (TreeStar, OR). Finally, cytokine responses to peptide pools in CD4 + and CD8 + T cells are represented following background subtraction (DMSO solvent condition).

### Analysis of antibody responses in serum

The antibody response against SHIV antigens was measured by an enzyme-linked immunosorbent assay (ELISA) using proteins from the NIH AIDS Research and reference Reagent Program (Env protein: gp120 Bal) or the NIBSC (Nef J5 and Gag rp27 proteins) as capture antigens. Anti-MV (Trinity Biotech) antibodies were detected by using commercial ELISA kits. Briefly, 1 μg/mL protein was used to coat a 96-well Nunc Maxisorp microtiter plate. Negative controls consisted of normal cynomolgus macaque serum and saturation assay buffer. The starting dilution of the sera was 1/50, and bound antibodies were detected with goat anti-monkey total Ig conjugated to horseradish peroxidase (Hrp) (Jackson Immunoresearch). Following TMB substrate addition, the optical density of the plates was read at 450 nm. Endpoint titers for each individual serum sample were calculated as the reciprocal of the last dilution giving twice the absorbance of the negative control sera. The detection limit of the ELISA was considered to be the starting dilution (1/50) of the test sera.

As described for the plasma antibodies, rectal secretion IgA binding antibodies were sought from fluid collected with Weck-Cel^TM^ sponges using goat anti-monkey IgA (Alpha Diagnostics, San Antonio, TX).

### Full hematology

Lymphocyte, eosinophils, and cell blood counts (CBC) were performed using a HMX A/L (Beckman Coulter).

### Virus-neutralization assays

Neutralization assays were performed as described previously^50^. Pseudovirus stocks were collected from the 293T cell supernatants at 48–72 h after transfection, clarified by centrifugation, divided into small volumes, and frozen at −80 °C. SHIV-SF162p3, HIV-1 SF162, and HIV-1 QH10, which are infectious viruses were propagated in activated human PBMCs. Fivefold serial dilutions of heat-inactivated serum samples were assayed for their inhibitory potential against the Env pseudoviruses using the TZM-bl indicator cell line, with luciferase as the readout as described. TZM-bl cells were plated and cultured overnight in flat-bottomed 96-well plates. A pseudovirus (2000 IU per well) in DMEM with 3.5% (vol/vol) FBS (Hyclone) and 40 μg/ml DEAE-dextran was mixed with serial dilutions of plasma or serum and subsequently added to the plated TZM-bl cells. At 48 h post-infection, the cells were lysed and luciferase activity was measured using a BioTek Synergy HT multimode microplate reader with Gen 5, v2.0 software. The average background luminescence from a series of uninfected wells was subtracted from each experimental well and infectivity curves were generated using GraphPad Prism v6.0d, where values from experimental wells were compared against a well containing a virus without a test reagent (100% infectivity). Neutralization IC_50_ titer values were calculated in GraphPad Prism v6.2 (GraphPad, San Diego, CA) using the dose-response inhibition analysis function with variable slope, log-transformed *x* values, and normalized *y* values.

### Statistical analysis

Kaplan–Meier curves and the log-rank Mantel–Cox test were used to test for differences in survival curves. Nonparametric Kruskal–Wallis and Dunn’s multiple comparisons tests were used to evaluate the immune responses obtained in the three different groups of immunization: MV, Wt, and Mt. The spearman rank correlation method was used for correlations. Statistical analyses were performed using GraphPad Prism v6.2 software (GraphPad, San Diego, CA).

### Reporting summary

Further information on research design is available in the Nature Research Reporting Summary linked to this article.

## Supplementary information

Supplementary Information

Reporting Summary

## Data Availability

The data that support the findings of this study are available from the authors on reasonable request; see author contributions for specific datasets.
