# Supplementary material for: A recombinant measles virus vaccine strongly reduces SHIV viremia and virus reservoir establishment in macaques
Source: NPJ Vaccines. 2021 Oct 22;6:123. doi: 10.1038/s41541-021-00385-6 (PMC8536681; doi:10.1038/s41541-021-00385-6)
Supplement: Supplementary file 1 — Supplementary Information [file 41541_2021_385_MOESM1_ESM.pdf]

Supplementary data:

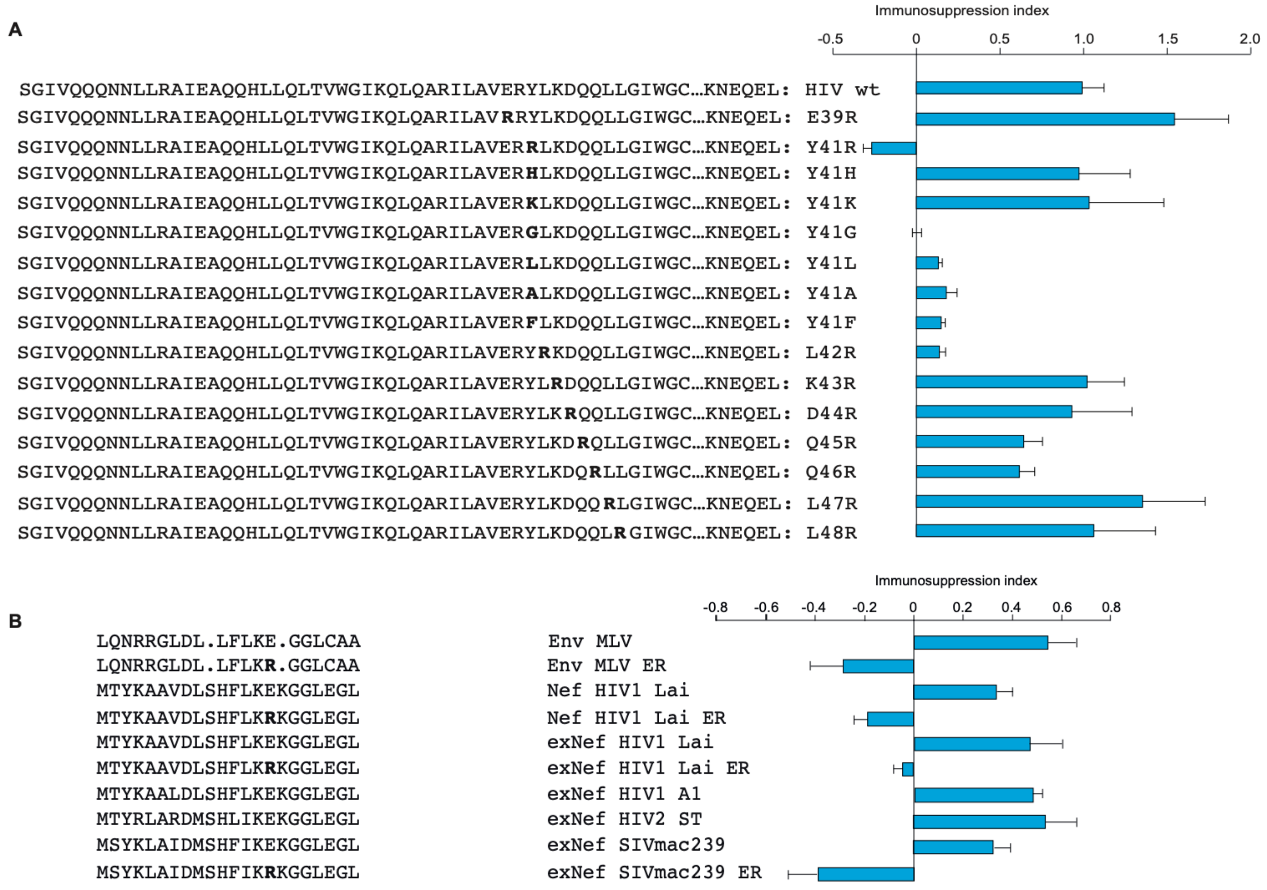

Supplementary Figure 1: Identification of HIV Env and SIV Nef IS domain mutations in tumor rejection assays

A. HIV Env. Left, wild-type and mutant sequences (mutated amino-acids are in bold) of HIV IS domain. Right, *in vivo* IS activity of Env wild-type and mutant proteins expressed in grafted tumor cells. Immunosuppressive indexes are based on the tumor size measurement of mice following s.c. injection of IS domain-expressing cells versus control cells. The Y41R mutation was selected for introduction in the MV-SHIV. **B. HIV and SIV Nef.** Left, conserved sequences of MLV Env (murine leukemia virus) and HIV-1, HIV-2, SIV Nef proteins with position of the selected E to R mutation indicated in bold. Right, *in vivo* IS activity of Nef wild-type and mutant proteins expressed in grafted tumor cells. exNef: excreted Nef: Nef protein is fused with a signal sequence resulting in its cellular export and lack of myristoylation. Mean values ± standard deviations (SD) are indicated.

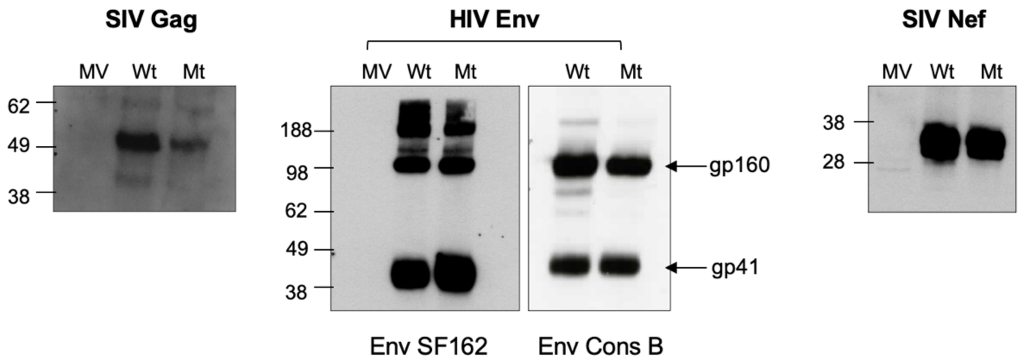

Supplementary Figure 2: Recombinant MV vectors expressing SHIV antigens.

Western blot analysis of SHIV proteins expression in cell lysates of Vero cells infected by MV or MV-SHIV viruses (MV: MV control, Wt: MV-SHIV Wt, Mt: MV-SHIV Mt). 2F12 monoclonal antibody was used for SIV Gag, F240 for HIV Env, MA1-71522 for SIV Nef.

Group	Time point	Pseudovirus								
Vaccine-controls	base line		BV165	BW695	CA117	CA870N	CBD006	BV834	CB135	CG393
		HIV-1 SF162	<16	<16	<16	<16	<16	<16	<16	<16
		SHIV162p3	<16	<16	<16	<16	<16	<16	<16	<16
		HIV-1 QH10	<16	<16	<16	<16	<16	<16	<16	<16
	post-boosts		BV165	BW695	CA117	CA870N	CBD006	BV834	CB135	CG393
		HIV-1 SF162	<16	<16	<16	<16	<16	<16	<16	<16
		SHIV162p3	<16	<16	<16	<16	<16	<16	<16	<16
		HIV-1 QH10	<16	<16	<16	<16	<16	<16	<16	<16
	post-challenges		BV165	BW695	CA117	CA870N	CBD006	BV834	CB135	CG393
		HIV-1 SF162	<16	<16	<16	<16	<16	<16	<16	<16
		SHIV162p3	<16	<16	<16	<16	<16	<16	<16	<16
		HIV-1 QH10	<16	<16	<16	<16	<16	<16	<16	<16
MV-SHIV wt	base line		BX409	BY250	BW430	CBE002	BZ509	CB637	BY549	BY791
		HIV-1 SF162	<16	<16	<16	<16	<16	<16	<16	<16
		SHIV162p3	<16	<16	<16	<16	<16	<16	<16	<16
		HIV-1 QH10	<16	<16	<16	<16	<16	<16	<16	<16
	post-boosts		BX409	BY250	BW430	CBE002	BZ509	CB637	BY549	BY791
		HIV-1 SF162	64	<16	<16	200	<16	<16	<16	<16
		SHIV162p3	<16	<16	<16	<16	<16	<16	<16	<16
		HIV-1 QH10	<16	<16	<16	<16	<16	<16	<16	<16
	post-challenges		BX409	BY250	BW430	CBE002	BZ509	CB637	BY549	BY791
		HIV-1 SF162	50	70	200	230	<16	<16	150	<16
		SHIV162p3	<16	<16	<16	<16	<16	<16	<16	<16
		HIV-1 QH10	<16	<16	<16	<16	<16	<16	<16	<16
MVSHIV IS Mt	base line		BW821	BX109	BX879	CG581	BV285	CBD005	CA142	CG889
		HIV-1 SF162	<16	<16	<16	<16	<16	<16	<16	<16
		SHIV162p3	<16	<16	<16	<16	<16	<16	<16	<16
		HIV-1 QH10	<16	<16	<16	<16	<16	<16	<16	<16
	post-boosts		BW821	BX109	BX879	CG581	BV285	CBD005	CA142	CG889
		HIV-1 SF162	<16	<16	<16	<16	<16	<16	<16	<16
		SHIV162p3	<16	<16	<16	<16	<16	<16	<16	<16
		HIV-1 QH10	<16	<16	<16	<16	<16	<16	<16	<16
	post-challenges		BW821	BX109	BX879	CG581	BV285	CBD005	CA142	CG889
		HIV-1 SF162	<16	250	<16	<16	<16	<16	250	<16
		SHIV162p3	<16	<16	<16	<16	<16	<16	<16	<16
		HIV-1 QH10	<16	<16	<16	<16	<16	<16	<16	<16

Supplementary Table 1: Neutralization activity of MV-SHIV-induced antibodies.

Assays of neutralizing activity in serum from vaccine-controls (measles virus, MV) or vaccinated macaques with MV-SHIV Wt or MV-SHIV Mt against HIV-1-SF162, SHIV162p3 or HIV-1 QHO pseudoviruses. Serums were collected at base line (pre-prime, week -2), post-boosts (4 weeks post-boost2, week +33), and post-challenge (3 weeks post first positive qRT-PCR, W+3 post infection). IC50: 50% inhibitory concentration, ND: not done.

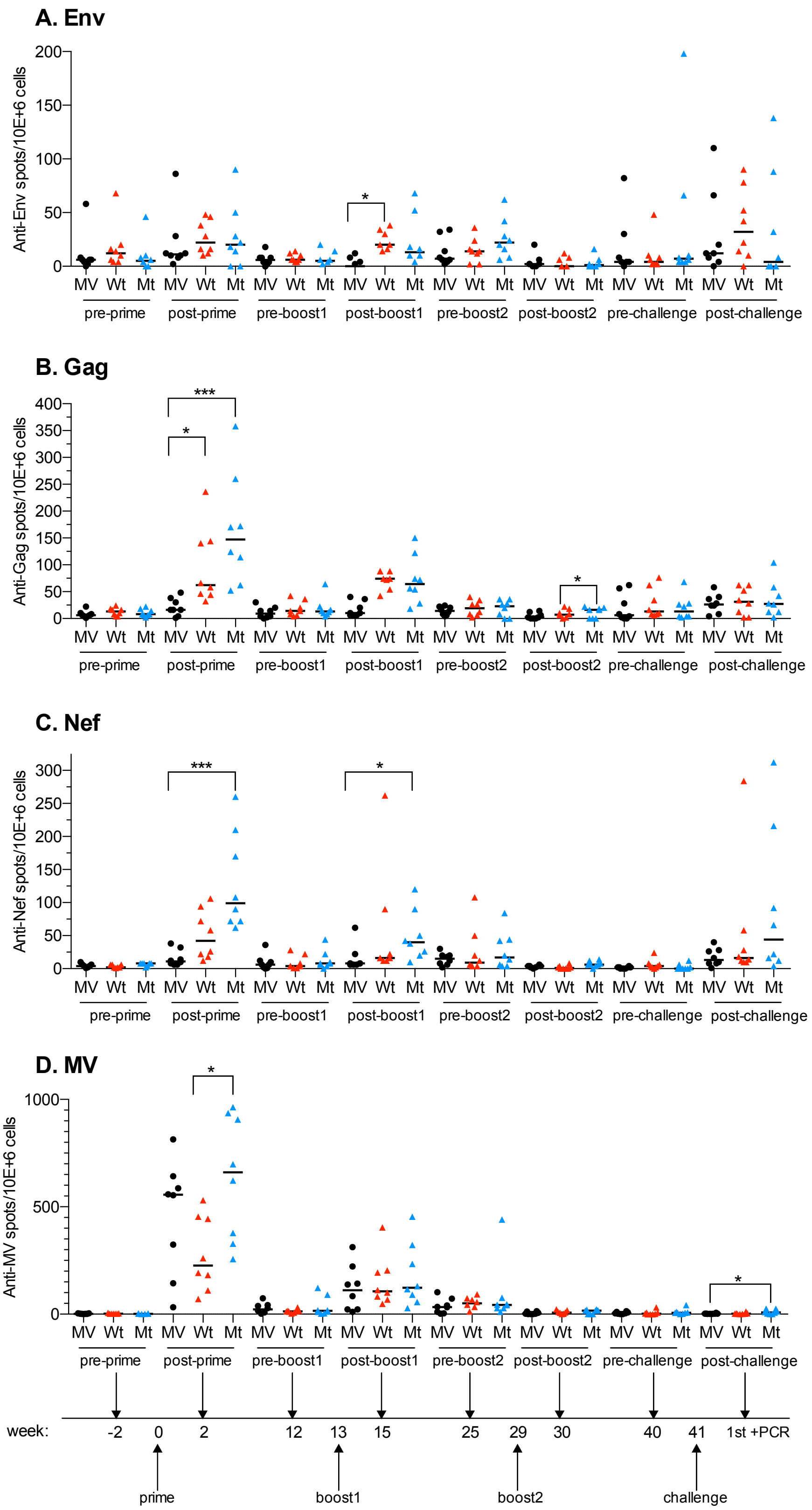

Supplementary Figure 3: longitudinal vaccine-elicited cellular immune responses.

A-D: Flispots assays (fluorospot assays), IFN-γ producing cells specific to (A) Env, (B) Gag, (C) Nef and (D) MV proteins. P values are calculated by the Kruskal-Wallis and Dunn's multiple comparisons tests. NS $p>0.05$, *$p<0.05$, **$p<0.01$, ***$p<0.001$. Group medians are plotted as horizontal line. Serum or PBMC were collected at pre-prime (week -2), post-prime (week +2), pre-boost1 (week +12), post-boost2 (week +15), pre-boost2 (week +25), post-boost2 (week +30), pre-challenge (week+40) and post-challenge (2 weeks post first positive qRT-PCR for SHIV162p3 RNA in plasma).

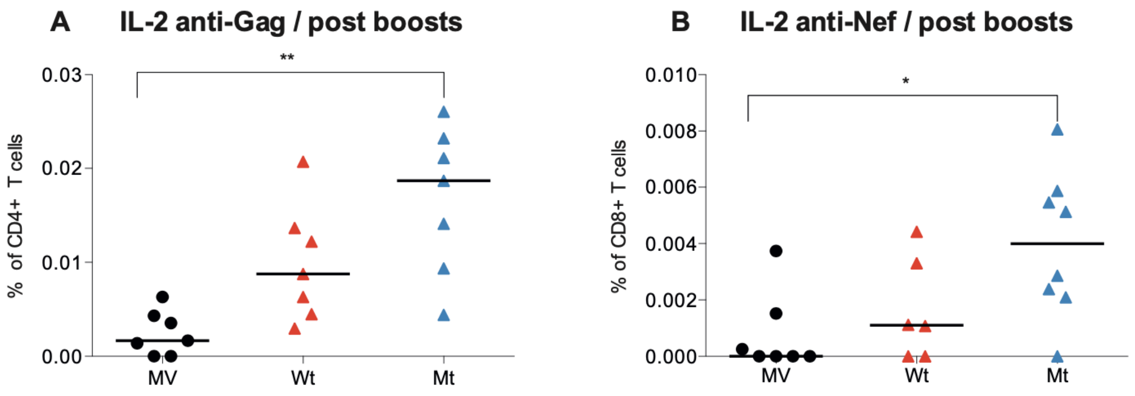

Supplementary Figure 4: Mutations of Nef and Env IS domains increase cellular immune responses. A. B. Vaccine-induced cellular responses analyzed post-boost 2 measured by IL2 intracellular staining (ICS) assays. MV indicates prime/boost with empty MV vector, Wt with MV-SHIV-Wt, and Mt with MV-SHIV-Mt. Statistical analyses are performed with the Kruskal-Wallis test and Dunn's multiple comparisons tests: NS p>0.05, *p<0.05, **p<0.01, ***p<0.001. Group medians are plotted as horizontal line.

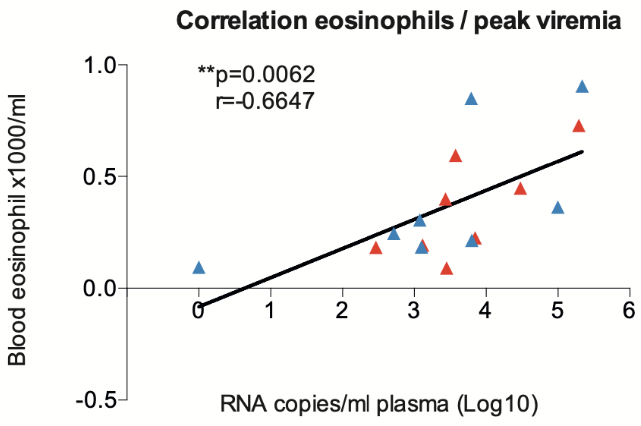

Supplementary Figure 5: Blood eosinophil counts correlated with plasma viremia in immunized animals (peak viremia).

For animals vaccinated with MV-SHIV Wt or Mt: correlation of the average eosinophil count over the course of the study (immunization and viral challenges) with peak viremia. P values reflect Spearman correlation non-parametric tests, two-tailed p-value. **p<0.01.

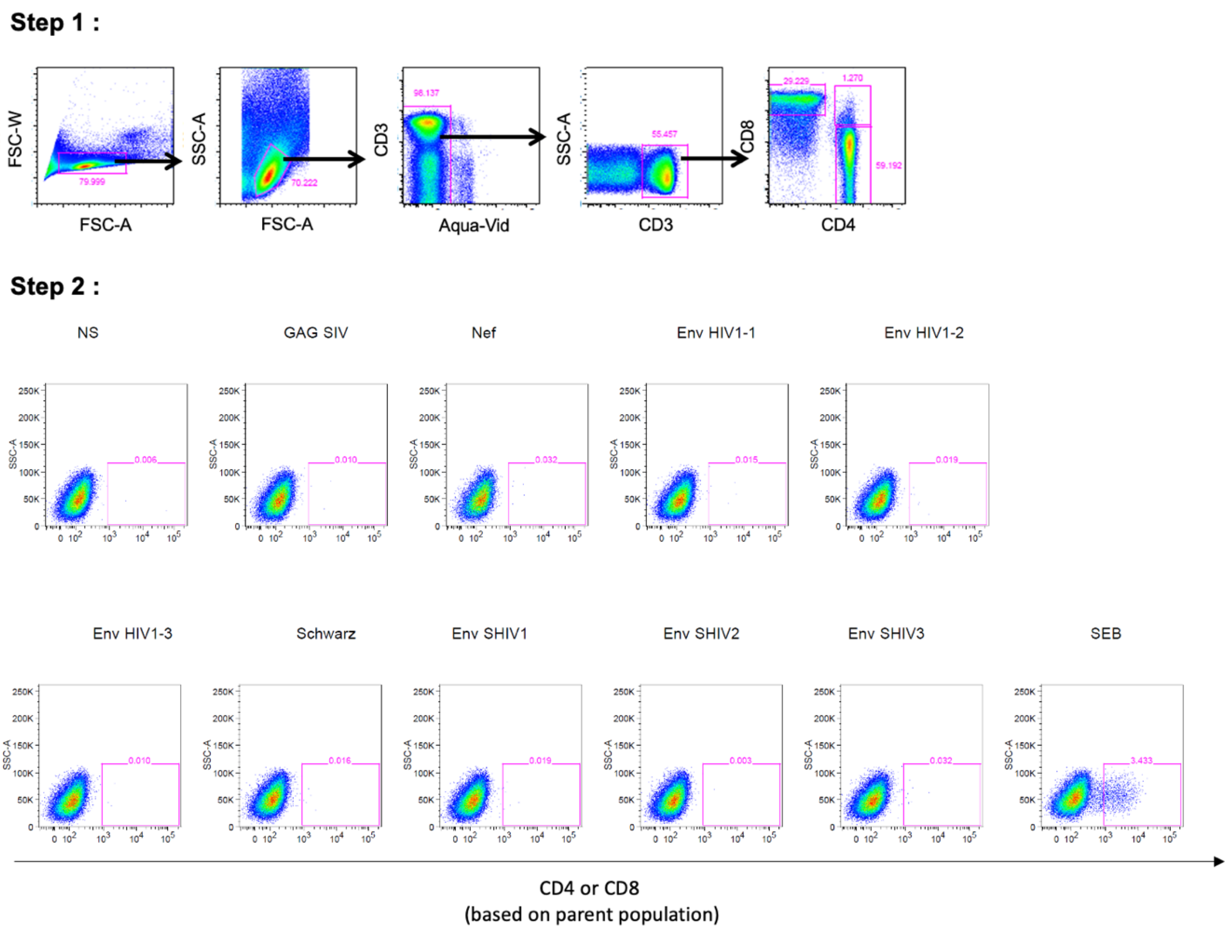

Supplementary Figure 6: Gating strategy.

Singlet were selected on FSC-W vs FSC-A gating. PBMC, and particularly lymphocytes, were selected on SSCA-A vs FSC-A gating; alive cells were selected as negative cells for Aqua-vid. T lymphocyte were selected based on their expression of CD3. T CD4, T CD8 and double positive cells were devised corresponding their expression of CD4 and CD8. Then for each population, expression of each marker was analyzed for all stimulations (IL2, IFNg, CD154 or TNF-a).
