# Supplementary material for: A recombinant measles virus vaccine strongly reduces SHIV viremia and virus reservoir establishment in macaques
Source: NPJ Vaccines. 2021 Oct 22;6:123. doi: 10.1038/s41541-021-00385-6 (PMC8536681; doi:10.1038/s41541-021-00385-6)
Supplement: Supplementary file 2 — Reporting Summary [file 41541_2021_385_MOESM2_ESM.pdf]

nature portfolio

Corresponding author(s): Thierry Heidmann & Frédéric Tangy

Last updated by author(s): Jul 9, 2021

Reporting Summary

Nature Portfolio wishes to improve the reproducibility of the work that we publish. This form provides structure for consistency and transparency in reporting. For further information on Nature Portfolio policies, see our Editorial Policies and the Editorial Policy Checklist.

Statistics

For all statistical analyses, confirm that the following items are present in the figure legend, table legend, main text, or Methods section.

n/a	Confirmed	
☐	☒	The exact sample size (*n*) for each experimental group/condition, given as a discrete number and unit of measurement
☐	☒	A statement on whether measurements were taken from distinct samples or whether the same sample was measured repeatedly
☐	☒	The statistical test(s) used AND whether they are one- or two-sided<br>*Only common tests should be described solely by name; describe more complex techniques in the Methods section.*
☒	☐	A description of all covariates tested
☒	☐	A description of any assumptions or corrections, such as tests of normality and adjustment for multiple comparisons
☐	☒	A full description of the statistical parameters including central tendency (e.g. means) or other basic estimates (e.g. regression coefficient) AND variation (e.g. standard deviation) or associated estimates of uncertainty (e.g. confidence intervals)
☐	☒	For null hypothesis testing, the test statistic (e.g. *F*, *t*, *r*) with confidence intervals, effect sizes, degrees of freedom and *P* value noted<br>*Give P values as exact values whenever suitable.*
☒	☐	For Bayesian analysis, information on the choice of priors and Markov chain Monte Carlo settings
☒	☐	For hierarchical and complex designs, identification of the appropriate level for tests and full reporting of outcomes
☐	☒	Estimates of effect sizes (e.g. Cohen's *d*, Pearson's *r*), indicating how they were calculated

Our web collection on statistics for biologists contains articles on many of the points above.

Software and code

Policy information about availability of computer code

Data collection: *Provide a description of all commercial, open source and custom code used to collect the data in this study, specifying the version used OR state that no software was used.*

Data analysis: *Provide a description of all commercial, open source and custom code used to analyse the data in this study, specifying the version used OR state that no software was used.*

For manuscripts utilizing custom algorithms or software that are central to the research but not yet described in published literature, software must be made available to editors and reviewers. We strongly encourage code deposition in a community repository (e.g. GitHub). See the Nature Portfolio guidelines for submitting code & software for further information.

Data

Policy information about availability of data

All manuscripts must include a data availability statement. This statement should provide the following information, where applicable:

- Accession codes, unique identifiers, or web links for publicly available datasets
- A description of any restrictions on data availability
- For clinical datasets or third party data, please ensure that the statement adheres to our policy

All data are available in the main text or supplementary materials.

Field-specific reporting

Please select the one below that is the best fit for your research. If you are not sure, read the appropriate sections before making your selection.

☒ Life sciences ☐ Behavioural & social sciences ☐ Ecological, evolutionary & environmental sciences

For a reference copy of the document with all sections, see nature.com/documents/nr-reporting-summary-flat.pdf

Life sciences study design

All studies must disclose on these points even when the disclosure is negative.

Sample size	In this study, we used 24 cynomolgus macaques divided into 3 groups of 8. The sample size was based on previous studies performed at IDMIT (Infectious Disease Models and Innovative Therapies) showing sufficient power analysis with 8 animals per group for vaccine immunity and viral challenges studies.
Data exclusions	No data of this study were excluded from the analysis.
Replication	All the samples taken on the NHP were unique, the analysis performed on these samples were done in triplicates when required.
Randomization	Animals were assigned into the 3 groups according to their MHC haplotypes so that each group contained a similar proportion of NHPs with equivalent haplotypes.
Blinding	The investigators responsible for collecting the samples and viral challenges were blinded, as well as those responsible for sample analysis. NHPs in the 3 groups were randomly assigned to the cages.

Reporting for specific materials, systems and methods

We require information from authors about some types of materials, experimental systems and methods used in many studies. Here, indicate whether each material, system or method listed is relevant to your study. If you are not sure if a list item applies to your research, read the appropriate section before selecting a response.

Materials & experimental systems

n/a	Involved in the study
☐	☒ Antibodies
☐	☒ Eukaryotic cell lines
☒	☐ Palaeontology and archaeology
☐	☒ Animals and other organisms
☒	☐ Human research participants
☒	☐ Clinical data
☒	☐ Dual use research of concern

Methods

n/a	Involved in the study
☒	☐ ChIP-seq
☐	☒ Flow cytometry
☒	☐ MRI-based neuroimaging

Antibodies

Antibodies used	Goat anti-monkey total Ig conjugated to horseradish peroxidase (Hrp) (Jackson Immunoresearch),<br>MA1-71522 for SIV Nef (Thermofisher Scientific),<br>2F12 for SIV Gag (USA NIH HIV reagent program,ARP-1610),<br>F240 for HIV gp41 (USA NIH HIV reagent program, ARP-7623),<br>Sheep anti-mouse IgG-horseradish peroxidase (HRP) conjugate (NA931V; GE Healthcare),<br>Sheep anti-human IgG-HRP conjugate (NA933V, GE Healthcare).
Validation	*Describe the validation of each primary antibody for the species and application, noting any validation statements on the manufacturer's website, relevant citations, antibody profiles in online databases, or data provided in the manuscript.*

Eukaryotic cell lines

Policy information about cell lines

Cell line source(s)	293T Human embryonic kidney cells (HEK, ATCC CRL -3216), HEK293T-NP helper cells (stably expressing MV-N and MV-P, Tangy F, EU patent 2006), African green monkey kidney cells (Vero, ATCC CCL-81).
Authentication	None of the cell lines used were authenticated.
Mycoplasma contamination	For all cell lines mycoplasma contamination testing status was routinely verified (# 30-1012K, ATCC).

Commonly misidentified lines (See ICLAC register) | N/A

Animals and other organisms

Policy information about studies involving animals; ARRIVE guidelines recommended for reporting animal research

Laboratory animals: 24 adult naïve cynommolgus macaques (Macaca fascicularis) male, age : 2-3 years

Wild animals: *Provide details on animals observed in or captured in the field; report species, sex and age where possible. Describe how animals were caught and transported and what happened to captive animals after the study (if killed, explain why and describe method; if released, say where and when) OR state that the study did not involve wild animals.*

Field-collected samples: *For laboratory work with field-collected samples, describe all relevant parameters such as housing, maintenance, temperature, photoperiod and end-of-experiment protocol OR state that the study did not involve samples collected from the field.*

Ethics oversight: The animals were used under the supervision of the veterinarians in charge of the animal facility. This study was approved and accredited under statement number A14-042 by the ethics committee "Comité d'Ethique en Expérimentation Animale du CEA" registered under number 44 by the French Ministry of Research.

Note that full information on the approval of the study protocol must also be provided in the manuscript.

Flow Cytometry

Plots

Confirm that:

☒ The axis labels state the marker and fluorochrome used (e.g. CD4-FITC).

☒ The axis scales are clearly visible. Include numbers along axes only for bottom left plot of group (a 'group' is an analysis of identical markers).

☒ All plots are contour plots with outliers or pseudocolor plots.

☒ A numerical value for number of cells or percentage (with statistics) is provided.

Methodology

Sample preparation: 2x10E6 PBMCs were incubated with anti-CD28 (1µg/mL) and anti-CD49d (1 µg/mL) (BD Biosciences, San Diego, CA, USA). Brefeldin A (Sigma-Aldrich, Saint-Louis, MO) were added at a final concentration of 10 µg/mL and plates were incubated at 37°C, 5% CO2 overnight and the different conditions for stimulation were applied. Cells were further stained with a viability dye (violet fluorescent reactive dye, Invitrogen), and then fixed and permeabilized with the BD Cytofix/Cytoperm reagent. Permeabilized cell samples were stored at -80°C before the staining procedure with the following antibodies: CD3, CD4 and CD8 (used as lineage markers), and IFN-g, TNF-a, IL2 and CD154 (BD Biosciences).

Instrument: BD Canto II Flow Cytometer (BD Biosciences).

Software: Flow Cytometry data were analyzed using Flowjo software (TreeStar, OR).

Cell population abundance: *Describe the abundance of the relevant cell populations within post-sort fractions, providing details on the purity of the samples and how it was determined.*

Gating strategy: Singlet were selected on FSC-W vs FSC-A gating. PBMC, and particularly lymphocytes, were selected on SSCA-A vs FSC-A gating; alive cells were selected as negative cells for Aquavid. T lymphocyte were selected based on their expression of CD3. T CD4, T CD8 and double positive cells were devised corresponding their expression of CD4 and CD8. Then for each population, expression of each marker were analyzed for all stimulations.

☒ Tick this box to confirm that a figure exemplifying the gating strategy is provided in the Supplementary Information.
